# Use of Nasopharyngoscopy Severity Classification of Chronic Epipharyngitis and Its Application for Evaluating the Treatment Outcomes of Epipharyngeal Abrasive Therapy

**DOI:** 10.7759/cureus.54067

**Published:** 2024-02-12

**Authors:** Yoshihiro Ohno

**Affiliations:** 1 Otolaryngology, Ohno ENT Clinic, Tokyo, JPN

**Keywords:** narrow-band image, zinc chloride, severity classification, epipharyngeal abrasive therapy, chronic epipharyngitis

## Abstract

Background

Chronic epipharyngitis causes postnasal drip, pharyngeal pain, pharyngeal discomfort, headache, and shoulder stiffness. Additionally, autonomic nervous system symptoms such as dizziness, general fatigue, and sleeplessness may occur. It can also contribute to the development of focal diseases. Although epipharyngeal abrasive therapy (EAT) is effective for chronic epipharyngitis involving the abrasion of the epipharynx with a zinc chloride solution, there is a lack of clear diagnostic criteria, and treatment outcomes are rarely reported.

Methodology

A classification of the severity of chronic epipharyngitis was attempted in 154 cases based on nasopharyngeal endoscopic findings, with a subsequent examination of treatment outcomes using EAT. Diagnosis of chronic epipharyngitis involved identifying redness, swelling, postnasal drip, and crusting of the epipharyngeal mucosa. Severity classification relied on a four-point scale measuring the degree of redness and swelling, with additional points assigned for the presence of postnasal drip and crusting. This classification also served as a criterion for judging treatment effectiveness. The prevalence and improvement rate of black spots and granular changes were assessed through nasopharyngeal endoscopy with narrow-band imaging. Subjective symptoms were evaluated using before and after treatment questionnaires, employing a four-point scale for symptoms commonly associated with chronic epipharyngitis (headache, postnasal drip, nasal obstruction, pharyngeal discomfort, pharyngeal pain, shoulder stiffness, tinnitus, ear fullness, dizziness, cough, and sputum). A 10-point numerical rating scale (NRS) was used to assess the physical condition.

Results

Following EAT, the severity of nasopharyngeal endoscopic findings notably improved, with a 76.0% (117/154) improvement rate (remarkable improvement: 19.5% (30), improvement: 56.5% (87)). The improvement rate for the chief complaint reached 85.7% (132/154), demonstrating significant enhancement in the score for each symptom. NRS scores also improved at a rate of 76.0% (117/154). A significant correlation was observed between the improvement in local findings and chief complaints. The prevalence of black spots and granular changes before EAT was 83.8% (129/154) and 64.3% (99/154), exhibiting improvement rates of 65.9% (87/132) and 54.8% (57/104), respectively.

Conclusions

Nasopharyngeal endoscopy proves valuable for diagnosing and assessing the severity of chronic epipharyngitis, as well as evaluating treatment effectiveness. The findings indicate that EAT is an effective treatment for chronic epipharyngitis, with improvements in local findings correlating with enhancements in the chief complaint. This underscores the importance of employing aggressive EAT in managing patients with chronic epipharyngitis.

## Introduction

Chronic epipharyngitis may go unnoticed if not specifically focused on as it presents with both local symptoms, such as postnasal drip, pharyngeal pain, sputum, and chronic cough, and varied symptoms, such as pharyngeal discomfort, headache, dizziness, and shoulder stiffness. Moreover, it can serve as an exacerbating factor for focal diseases such as palmoplantar pustulosis and immunoglobulin A nephropathy [1,2]. While nasopharyngeal endoscopy may reveal local inflammatory findings for diagnosis, clear diagnostic criteria and severity evaluation are lacking. Epipharyngeal abrasive therapy (EAT) [3], involving the abrasion of the epipharyngeal mucosa with nasal swabs soaked in a zinc chloride solution or a pharyngeal swab, is considered an effective treatment [4-9]. However, there are limited reports on its outcomes. This study aimed to diagnose and categorize the severity of chronic epipharyngitis based on nasopharyngeal endoscopic findings and assess the results of EAT. Additionally, the study investigated the prevalence and improvement rate of black spots and granular changes [3], characteristic features observed through narrow-band imaging (NBI) during nasopharyngeal endoscopy in chronic epipharyngitis.

## Materials and methods

Subjects

This study included 154 patients, 34 men and 120 women aged 14-85 years (mean age = 51.8 years; standard deviation = 16.7 years), diagnosed with chronic epipharyngitis and treated with EAT between April 2019 and December 2020. The diagnosis of chronic epipharyngitis was based on the suspicion of the disease arising from symptoms and nasopharyngeal endoscopic findings, which included redness, swelling, postnasal drip, and crusting of epipharyngeal origin. Acute epipharyngitis cases within two to three weeks of onset were excluded.

This study was approved by the Ethical Review Committee of the Japan Medical Association (approval number: 30-14), and informed consent was obtained from all patients.

Topical therapy

After spraying the epipharynx with 3,000 times adrenaline + 4% lidocaine solution as local anesthesia, EAT was performed by abrading the epipharyngeal mucosa from the nasal cavity with a nasal swab soaked in 1% zinc chloride solution and from the oral cavity with a pharyngeal swab. Nasal nebulization, consisting of cefmenoxime hydrochloride, dexamethasone sodium phosphate, and adrenaline, was administered after EAT. Treatment efficacy was assessed following 10 EAT sessions as a standard protocol. Patients were instructed to lavage the epipharynx with a saline solution, following Hotta’s method [10]. Additional treatments included nasal drops with Misatol Rhino Lotion® (a plum extract) and mouth tapes to prevent mouth breathing, both based on Hotta’s method [10]. The “Ka-iube exercise” was also performed for those who requested it. Nasal drops with Misatol Rhino Lotion were used in 10 patients, and mouth tapes were used in 12 patients.

Nasopharyngeal endoscopic evaluation of epipharyngeal findings

**Figure 1:**
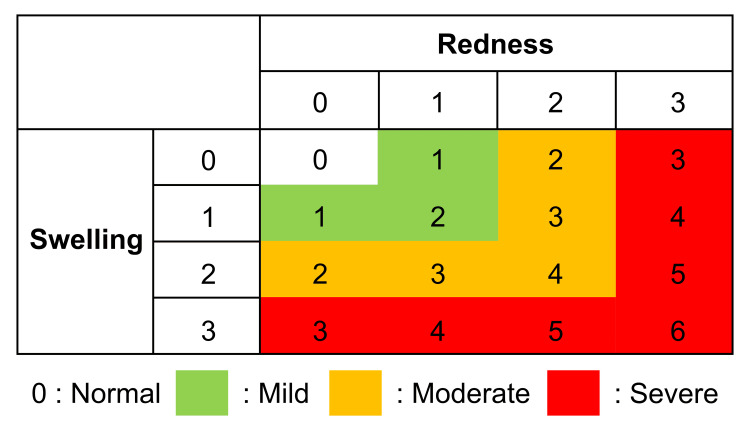
Classification of severity of local findings. The severity was classified as shaded with priority given to the degree of redness and swelling. Points were added if crusting or postnasal drip was observed (++: 2 +: 1 −: 0). In cases where points were added, the total score was used to determine the severity of the disease. 1 or 2 points: mild cases; 3 or 4 points: moderate cases; 5 or more points: severe cases.

**Figure 2:**
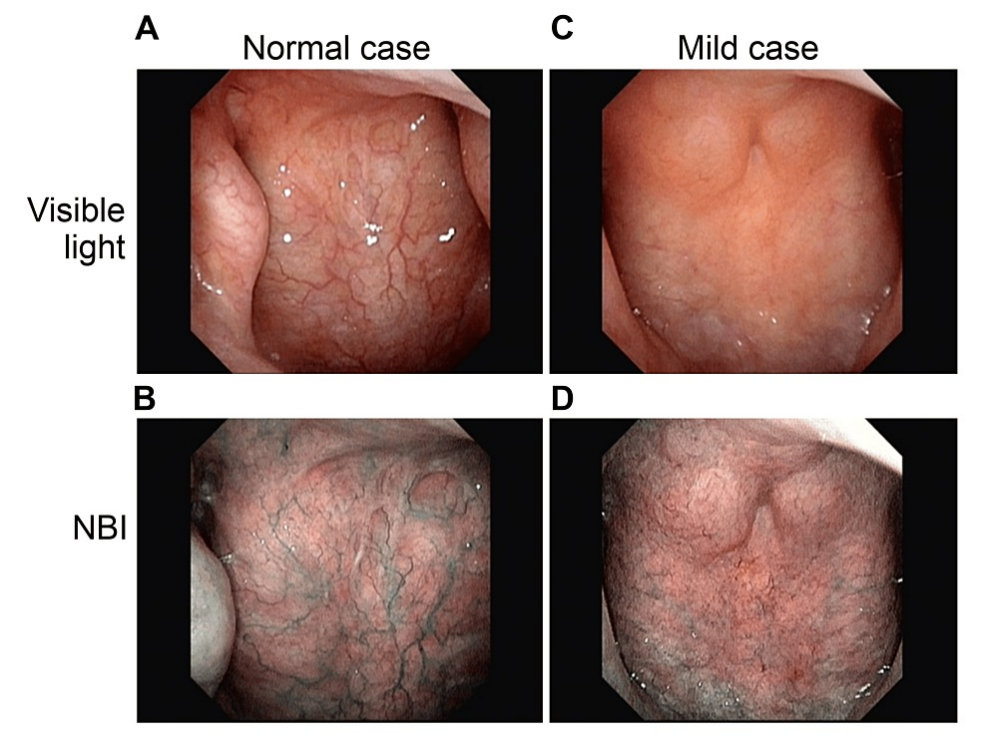
Examples of severity classification (normal and mild cases). A and B: normal case, redness: 0, swelling: 0, total score: 0. C and D: mild case, redness: 1, swelling: 1, total score: 2. A and C: visible light. C and D: narrow-band image (NBI).

**Figure 3:**
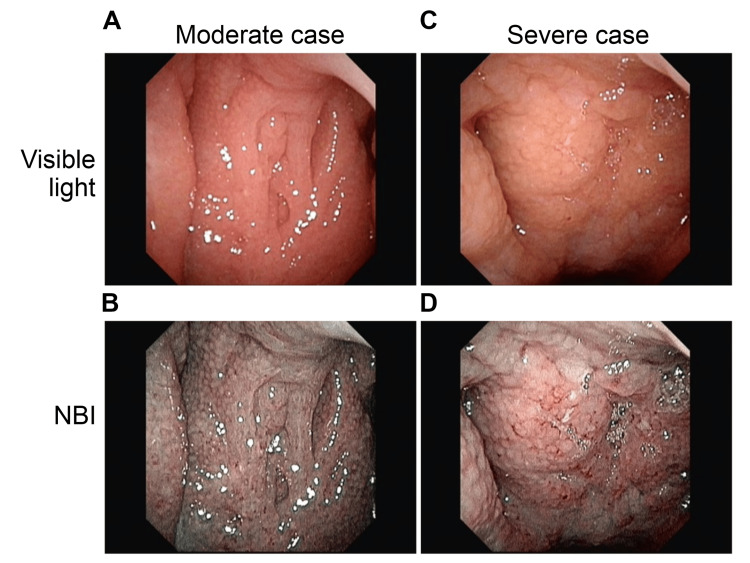
Examples of severity classification (moderate and severe cases). A and B: moderate case, redness: 2, swelling: 2, total score: 4. C and D: severe case, redness: 2, swelling: 3, postnasal drip: 1, total score: 6. A and C: visible light. C and D: narrow-band imaging (NBI).

Before initiating treatment, a nasopharyngeal endoscope (Olympus ENF-V3) was employed to assess the epipharyngeal mucosa through video capture of static images (normal light and NBI) saved on a personal computer. The degree of redness and swelling of the epipharyngeal mucosa was rated on a four-level scale (severe: 3, moderate: 2, mild: 1, none: 0). If postnasal drip or crusting was observed, an additional three-level score was assigned (++: 2, +: 1, −: 0), and the total score was computed. Severe cases were defined as having severe redness or swelling with a total score of 5 or more, moderate cases as having moderate redness or swelling with a total score of 3 or 4, and mild cases as having mild redness or swelling with a total score of 1 or 2. A total score of 0 after treatment was considered normal. The severity classification is shown in Figure 1. As a pretreatment evaluation, endoscopic findings of normal cases as controls and cases judged as mild to severe are shown in Figure 2 and Figure 3.

The prevalence and improvement rates for black spots and granular changes of the epipharyngeal mucosa in NBI were also evaluated with three-level scores (++: 2, +: 1, −: 0). Improvement was defined as a decrease in score after treatment.

**Figure 4:**
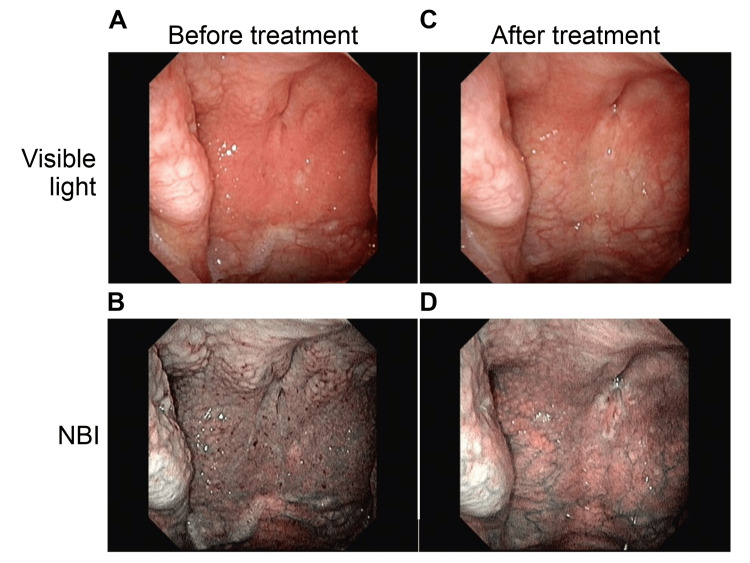
Example of evaluation of treatment efficacy by severity classification. A and B: before treatment, severe classification with redness: 3, swelling: 2, postnasal drip: 1, total score: 6. C and D: after treatment, mild classification with redness: 1, swelling: 1, total score: 2. A and C: visible light. C and D: narrow-band imaging (NBI). Severe to mild classification was evaluated as a remarkable improvement. NBI also shows improvement in black spots and granular changes after treatment.

After treatment, static images of the epipharyngeal mucosa were stored in the same manner as before treatment, and the same severity classification was employed. The effect of treatment on local findings was evaluated as follows: remarkable improvement: severe → mild or severe/moderate/mild → normal; improvement: change from severe to moderate or from moderate to mild; no change: no change in severity; and worsening: worsened severity. An example of remarkable improvement from severe to mild is shown in Figure 4.

Cases with remarkable improvement and those with improvement were classified as improvement, and the improvement rate was calculated.

Evaluation of the subjective symptoms

**Figure 5:**
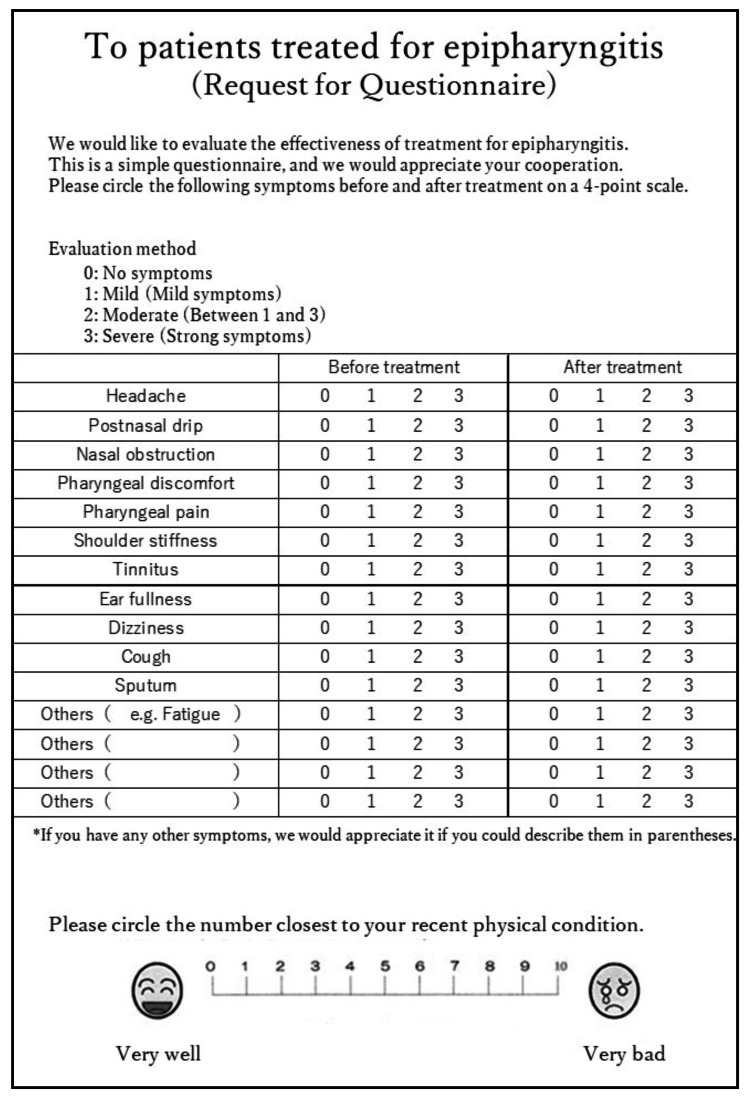
Patient questionnaire form. Each symptom that is likely to be perceived in chronic epipharyngitis was described in advance. Other symptoms were separately written in the lower part of the form, and each was scored on a four-point scale (severe: 3, moderate: 2, mild: 1, no symptoms: 0). The numerical rating scale for physical condition, shown in the lower part of the figure, used a 10-point score filled in before and after treatment. This questionnaire is developed by the author.

Patients were requested to complete a questionnaire (Figure 5) regarding their subjective symptoms before and after treatment. The questionnaire included a list of symptoms commonly associated with chronic epipharyngitis (headache, postnasal drip, nasal obstruction, pharyngeal discomfort, pharyngeal pain, shoulder stiffness, tinnitus, ear fullness, dizziness, cough, and sputum). These symptoms were rated on a four-point scale (severe: 3, moderate: 2, mild: 1, and no symptoms: 0). Patients were also encouraged to report any unlisted symptoms. The chief complaints and improvement of each subjective symptom were judged as follows: remarkable improvement: 3 to 1 or 3, 2, 1 to 0; improvement: 3 → 2 or 2 → 1; no change: no change in the score; and worsening: worsened score.

The chief complaints were obtained from patient interviews, and the improvement rate of each item was examined. The improvement rates of the chief complaints were evaluated as “remarkable improvement” or “improvement.” For each subjective symptom score, the improvement rate was calculated for each item with one or more statements before and after treatment, and the improvement of the score was statistically examined.

A numerical rating scale (NRS) for physical condition was written at the bottom of the questionnaire (Figure 5), and the patients were asked to fill in the NRS before and after treatment. The determination of the improvement in NRS was made as follows: remarkable improvement: improvement of three or more steps (e.g., from 8 to 5) or 0; improvement: improvement of one or two steps; no change: no change in the score; and worsening: worsened score.

For physical condition, the improvement rate was calculated as remarkable improvement and improvement. The improvement of scores before and after treatment was statistically examined.

Relationship between improvement in local findings and improvement in chief complaint and physical condition

The relationship between improvement in local findings and improvement in chief complaints and physical condition was statistically examined for items of remarkable improvement, other, greater than or equal to improvement, and their combinations.

Statistical methods

Wilcoxon signed-rank test was employed to analyze the improvements in subjective symptoms and physical condition scores, while Fisher’s exact test was used to assess the association between local findings and improvements in chief complaints and physical condition. Statistical analysis was conducted using EZR (easy R).

## Results

Local findings, severity classification, and treatment outcomes

**Figure 6:**
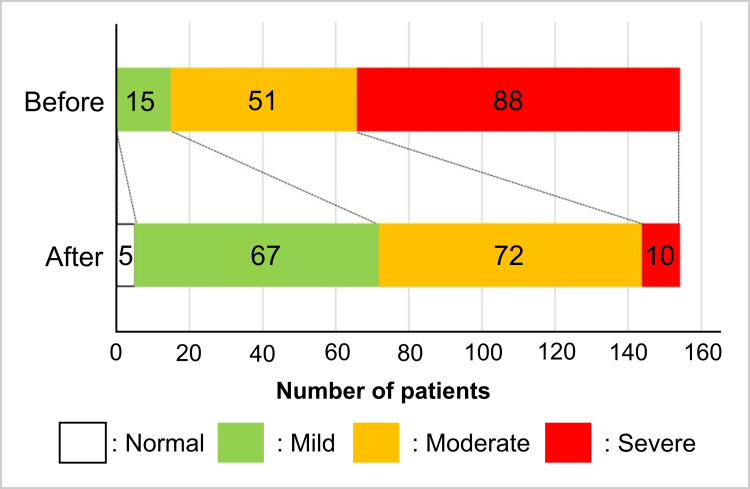
Local findings severity classification before and after treatment. Number of patients in the severity classification. Before treatment: normal: 0, mild: 15, moderate: 51, and severe: 88. After treatment: normal: 5, mild: 67, moderate: 72, and severe: 10. The severity of local findings became milder after treatment. The rate of improvement of local findings severity was 76.0% (a remarkable improvement of 19.5%).

The pretreatment severity in this local findings’ severity classification was severe in 88 (57.1%), moderate in 51 (33.1%), and mild in 15 (9.7%) patients. After treatment, the disease exhibited milder manifestations, with 10 (6.5%) classified as severe, 72 (46.8%) as moderate, 67 (43.5%) as mild, and 5 (3.2%) as normal (Figure 6). The improvement rate of local findings was 76.0%, with 30 (19.5%) showing remarkable improvement and 87 (56.5%) showing improvement. The number of patients with no change and worsening score was 34 (22.1%) and 3 (1.9%), respectively.

Improvement in subjective symptoms

The improvement rate of chief complaints was 85.7%, with 85 (55.2%) showing remarkable improvement, 47 (30.5%) showing improvement, 22 (14.3%) showing no change, and 0 (0%) showing worsening. A list of the chief complaints and their respective improvement rates is shown in Table 1. Postnasal drip was the most common complaint (29 cases), followed by pharyngeal discomfort (24 cases), pharyngeal pain (20 cases), cough (15 cases), sputum (14 cases), and hoarseness (10 cases), with various symptoms as chief complaints, as shown in Table 1.

**Table 1:** List of chief complaints and improvement rate.

Symptoms of chief complaint	n	Improvement rate, %
Postnasal drip	29	82.8
Pharyngeal discomfort	24	83.3
Pharyngeal pain	20	90.0
Cough	15	93.3
Sputum	14	85.7
Hoarseness	10	100.0
Dizziness	8	62.5
Skin eruption	7	85.7
Shoulder stiffness	6	100.0
Headache	4	75.0
Nasal obstruction	3	100.0
Ear fullness	3	33.3
Fatigue or malaise	3	100.0
Ear pain	2	100.0
Tinnitus	2	50.0
Mild fever	2	100.0
Bad smell in the nose	1	100.0
Neck pain	1	100.0
Total	154	85.7

The improvement rate of the NRS indicating physical condition was 76.0%, with 74 (48.1%) showing remarkable improvement, 43 (27.9%) showing improvement, 23 (14.9%) showing no change, and 14 (9.1%) showing worsening.

Table 2 shows significant improvement in each subjective symptom score after treatment. All symptoms that had been indicated on the questionnaire in advance were significantly improved (p < 0.001). Regarding other symptoms, hoarseness (p < 0.001), fatigue or malaise (p = 0.0016), psychological symptoms (p = 0.0074), and skin eruptions (p = 0.0069) showed significant improvement. Of the 11 patients with psychological symptoms, six had sleep disorders, two had panic disorders, and one each had anxiety, poor memory, and poor concentration. The 10 skin eruptions included six cases of palmoplantar pustulosis, one case each of atopic dermatitis and psoriasis, and two cases of other skin eruptions.

**Table 2:** Improvement rate of chief complaint and each subjective symptom score after treatment and results of statistical analysis. *: symptoms not listed on the questionnaire previously.

Symptoms	n	Improvement rate (remarkable), %	P-value
Chief complaint	154	85.7 (55.2)	<0.001
Headache	94	72.3 (50.0)	<0.001
Postnasal drip	127	67.7 (35.4)	<0.001
Nasal obstruction	105	70.5 (46.7)	<0.001
Pharyngeal discomfort	133	74.4 (57.9)	<0.001
Pharyngeal pain	112	77.7 (57.1)	<0.001
Shoulder stiffness	117	76.1 (47.0)	<0.001
Tinnitus	62	58.1 (45.2)	<0.001
Ear fullness	57	68.4 (61.4)	<0.001
Dizziness	60	70.0 (61.7)	<0.001
Cough	93	72.0 (49.5)	<0.001
Sputum	128	64.1 (46.9)	<0.001
Hoarseness*	21	85.7 (61.9)	<0.001
Fatigue or malaise*	14	92.9 (71.4)	0.0016
Psychological symptoms*	11	81.8 (45.5)	0.0074
Skin eruption*	10	90.0 (40.0)	0.0069

Relationship between local findings and improvement in chief complaints

When examining the relationship between the improvement of local findings and chief complaints, significant correlations were found between cases where local findings and chief complaints were remarkably improved and others (p = 0.013), local findings improved or more and the chief complaint improved remarkably (p = 0.002), and local findings and chief complaints improved or more and others (p < 0.001) (Table 3). No significant correlation was found between local findings and improvement in the NRS, indicating a general condition.

**Table 3:** Association between local findings and rate of improvement of chief complaints. The table shows the number of patients with remarkable improvement (RM) and improvement or more (IM), and other groups (Others) with respect to chief complaint (CC) and local findings (LF). Significant differences are shown in each association. Fisher’s exact test was used for statistical analysis.

	CC RM	Others
LF RM	23	7
Others	62	62
		p = 0.013
	CC RM	Others
LF IM	73	44
Others	12	25
		p = 0.002
	CC IM	Others
LF IM	107	10
Others	25	12
		p < 0.001

Prevalence and improvement rate of local findings in NBI

In NBI, black spots were present in 129 (83.8%) patients before treatment (++35 (22.7%), +94 (61.0%), and −25 (16.2%)). In three cases, black spots were not observed until after treatment. The improvement rate after treatment was 65.9% (87/132). Granular changes were observed in 99 (64.3%) patients (++18 (11.7%), +81 (52.6%), and −55 (35.7%)). In five cases, no granular changes were observed until after treatment. The improvement rate after treatment was 54.8% (57/104). Black spot and granular change scores significantly improved after treatment (p < 0.001).

## Discussion

The concept of chronic epipharyngitis was initially introduced by Yamazaki [11] and Horiguchi [12] in the homework report of the Japanese Otorhinolaryngological Society in the 1960s. Horiguchi reported numerous therapeutic effects and mechanisms of action of zinc chloride treatment on the epipharyngeal mucosa [13]. However, despite these findings, treatment for chronic epipharyngitis has not gained widespread acceptance and has been practiced sparingly by some otolaryngologists. In 2010, Hotta [14], a nephrologist, speculated that EAT for chronic epipharyngitis is effective for IgA nephropathy and other focal diseases, as well as various other cases. Since then, EAT for chronic epipharyngitis has regained attention.

While there have been no clear diagnostic criteria for chronic epipharyngitis, Horiguchi [12] made a diagnosis based on the degree of bleeding and pain caused by the procedure and the smear preparation of an epipharyngeal abrasion specimen. However, the degree of bleeding and pain is greatly influenced by the intensity of the procedure, making it impractical to prepare epipharyngeal abrasion smears for all patients. With the recent development of endoscopes, especially the widespread use of electronic endoscopes, it has become possible to observe local findings in the epipharynx with high resolution. In this study, the author attempted to diagnose and classify the severity of chronic epipharyngitis based on nasopharyngeal endoscopic findings. The diagnosis was made in cases where redness and swelling of the epipharyngeal mucosa, postnasal drip, and crusts of epipharyngeal origin were observed, and cases that appeared to be acute epipharyngitis within two to three weeks of disease onset were excluded. The severity of epipharyngitis was classified based on the degree of redness and swelling seen in patients with chronic epipharyngitis, which were rated on a four-point scale from severe to none, with additional points given when postnasal drip and crusts were observed. The improvement rate was evaluated using severity classification before and after treatment. Approximately 80% of patients were expected to improve, as in previous studies [4-6,15]. This study suggests the effectiveness of EAT for this disease, and aggressive treatment, including EAT, should be performed for patients with chronic epipharyngitis.

Hotta [1,15] identified the following three mechanisms of action in the pathogenesis of the various subjective symptoms of chronic epipharyngitis: (1) symptoms caused by epipharyngitis itself or by the dissipation of inflammation, (2) symptoms caused by disturbances of the neuroendocrine system (especially the autonomic nervous system), and (3) secondary diseases due to autoimmune abnormalities. In previous studies [4-6], postnasal drip was the most frequent chief complaint of the condition in (1). Chronic sinusitis is a typical disease that causes persistent postnasal drip; however, the presence of chronic epipharyngitis should be considered when a patient complains of postnasal drip despite the absence of sinusitis on imaging and other findings. Other symptoms, such as pharyngeal discomfort, pharyngeal pain, cough, sputum, headache, and shoulder stiffness, are often noticed, and significant improvement of symptoms was observed with EAT in this study. The pathophysiology of (2) is thought to be a disturbance of the autonomic nervous system caused by chronic epipharyngitis. The epipharynx is rich in C fibers, which are sensory nerves of the vagus nerve. Inflammation of the epipharynx turns off the ventral vagal system among the vagus nerves, resulting in a state of sympathetic overactivity, which is believed to cause autonomic imbalance [16]. Among the symptoms that significantly improved in this study, dizziness, fatigue, malaise, and mental symptoms (including insomnia) can be attributed to this condition. The epipharynx is a physiological site of inflammation where activated lymphocytes are abundant even in healthy subjects [14], suggesting a relationship between chronic epipharyngitis and autoimmune diseases. In this study, improvement of skin eruptions, including palmoplantar pustulosis and atopic dermatitis, was also observed.

The mechanisms of action of EAT on chronic epipharyngitis include the following: (1) the stringent anti-inflammatory effect of zinc chloride, (2) the blood-letting effect of EAT, and (3) the neuromodulation effect activated through stimulation of the vagus nerve [1,15]. Mechanism (1) can be seen as a direct anti-inflammatory effect of rubbing the epipharynx with zinc chloride, and zinc also exhibits bactericidal and antiviral effects [1,15]. Mechanism (2) may be related to the fact that when EAT is performed on patients with chronic epipharyngitis, most cases are accompanied by bleeding. In chronic epipharyngitis induced by chronic antigenic stimuli, such as bacteria, viruses, and dust, the immune system and microvessels, capillary lymphatics, and vagus nerve are dysfunctional, and venous congestion is observed [1,15]. Black spots on NBI indicate bleeding or severe congestion of the mucosal surface layer, which is highly correlated with severe bleeding during epipharyngeal abrading [3]. The perivascular lumen around cerebral arterioles functions as a functional lymphatic vessel and drains extracranially via the cerebral lymphatic drainage pathway [17,18]. The pharyngeal lymphatic vessels are the key to the extracranial cerebral lymphatic drainage pathway, and it has been suggested that mechanical phlebotomy by EAT reduces venous and lymphatic congestion in the epipharynx, resulting in functional recovery of the cerebral lymphatic drainage pathway and possibly improving cerebral circulation and cerebral neuron function [1,15]. EAT also suppresses the production of inflammatory cytokines interleukin-6 and tumor necrosis factor-alpha in the epipharyngeal mucosa [19]. Mechanism (3) speculates that EAT on the epipharyngeal mucosa, which is rich in vagus nerve fibers, can be a vagus nerve stimulation treatment [1,15]. It is speculated that the sympathetic hyperactivity condition caused by chronic epipharyngitis has improved. To enhance the effects of mechanisms of action (2) and (3) the entire epipharyngeal mucosa should be abraded with appropriate strength as much as possible.

In the present study, the author investigated the prevalence and improvement rate of black spots and granular changes in NBI. Mogitate et al. [9] reported a 73% prevalence of black spots and a 76% prevalence of granular changes. This study found prevalence rates of 84% and 64%, respectively. As mentioned earlier, black spots are indicative of venous congestion in the epipharyngeal mucosa and are expected to improve with EAT’s phlebotomy effect, showing an improvement rate of approximately 66%. Granular changes, associated with venous capillary network dilatation and interstitial edema, tend to disappear with EAT [3], demonstrating an improvement rate of around 55% in our study. While NBI is not widely utilized in otolaryngology clinics currently, the author suggests considering its incorporation into the severity assessment of chronic epipharyngitis as its usage becomes more widespread in the future.

The Epipharyngeal Abrasive Therapy Study Committee was established to evaluate the severity of EAT for chronic epipharyngitis and its therapeutic effect [15,20], and analysis of the results is expected in the future. The committee’s evaluation items included (A) color tone; (B) swelling; (C) mucus, crusts, and postnasal drip in endoscopic findings; and (D) bleeding during abrasion. In the original draft, each of these was rated on a four-point scale of normal, mild, moderate, and severe. However, when the committee members tried to evaluate the endoscopic findings, they found considerable variation; therefore, at present, the committee is proceeding with a three-point scale of normal, mild to moderate, and severe. Evaluating epipharyngeal mucosal inflammation findings by nasopharyngeal endoscopy is difficult unless a considerable number of cases are experienced. In this study, bleeding was excluded from the evaluation, but most of the bleeding was eliminated or improved by EAT (improvement rate 92.2%) when the cases were evaluated in the above three levels. All patients with severe bleeding before treatment (85 patients) showed improvement. While the degree of bleeding aids in assessing its severity before treatment, it must be excluded when determining EAT efficacy.

The primary limitation of this study is that it was conducted at a single institution for research and evaluation. Therefore, a multicenter study that confirms the present findings, such as the Epipharyngeal Abrasive Therapy Study Committee, should be conducted.

## Conclusions

In my previous studies, nasopharyngeal endoscopic findings were useful in diagnosing and evaluating the severity of chronic epipharyngitis, suggesting the effectiveness of EAT for this condition. The findings indicate that EAT is an effective treatment for chronic epipharyngitis, with improvements in local findings correlating with enhancements in the chief complaint. I hope that nasopharyngeal endoscopy will be performed in patients with symptoms of chronic epipharyngitis and that aggressive treatment, including EAT, will be performed in patients with inflammatory findings.
